# Merging metagenomics and geochemistry reveals environmental controls on biological diversity and evolution

**DOI:** 10.1186/1472-6785-14-16

**Published:** 2014-05-28

**Authors:** Eric B Alsop, Eric S Boyd, Jason Raymond

**Affiliations:** 1 School of Earth and Space Exploration, Arizona State University, ISTB4, Room 795, 781 E. Terrace Rd, Tempe, AZ 85287, USA; 2 Department of Microbiology and Immunology and the Thermal Biology Institute, Montana State University, 109 Lewis Hall, Bozeman, MT 59717, USA; 3 Wisconsin Astrobiology Research Consortium, University of Wisconsin, Weeks Hall, Madison, WI 53706, USA

**Keywords:** Metagenomics, Microbial ecology, Hydrothermal ecosystems, Geochemistry, Markov clustering

## Abstract

**Background:**

The metabolic strategies employed by microbes inhabiting natural systems are, in large part, dictated by the physical and geochemical properties of the environment. This study sheds light onto the complex relationship between biology and environmental geochemistry using forty-three metagenomes collected from geochemically diverse and globally distributed natural systems. It is widely hypothesized that many uncommonly measured geochemical parameters affect community dynamics and this study leverages the development and application of multidimensional biogeochemical metrics to study correlations between geochemistry and microbial ecology. Analysis techniques such as a Markov cluster-based measure of the evolutionary distance between whole communities and a principal component analysis (PCA) of the geochemical gradients between environments allows for the determination of correlations between microbial community dynamics and environmental geochemistry and provides insight into which geochemical parameters most strongly influence microbial biodiversity.

**Results:**

By progressively building from samples taken along well defined geochemical gradients to samples widely dispersed in geochemical space this study reveals strong links between the extent of taxonomic and functional diversification of resident communities and environmental geochemistry and reveals temperature and pH as the primary factors that have shaped the evolution of these communities. Moreover, the inclusion of extensive geochemical data into analyses reveals new links between geochemical parameters (e.g. oxygen and trace element availability) and the distribution and taxonomic diversification of communities at the functional level. Further, an overall geochemical gradient (from multivariate analyses) between natural systems provides one of the most complete predictions of microbial taxonomic and functional composition.

**Conclusions:**

Clustering based on the frequency in which orthologous proteins occur among metagenomes facilitated accurate prediction of the ordering of community functional composition along geochemical gradients, despite a lack of geochemical input. The consistency in the results obtained from the application of Markov clustering and multivariate methods to distinct natural systems underscore their utility in predicting the functional potential of microbial communities within a natural system based on system geochemistry alone, allowing geochemical measurements to be used to predict purely biological metrics such as microbial community composition and metabolism.

## Background

The taxonomic and metabolic compositions of microbial communities are both shaped and constrained by the characteristics of their local environment. The characteristics of an environment, in turn, are defined by dynamic physical, geochemical and biological components whose complex interactions are very seldom included in –omics-enabled interrogations of natural communities. This is despite the fact that several recent studies, typically focusing on only a few easily measured environmental parameters, show that natural communities are very tightly tuned—both in overall metabolic function and in community population structure—to nuances of their environment [1-3]. The architecture of natural communities is dictated by competitive and facilitative interactions that function to mold the metabolic strategies responsible for deriving energy and nutrients and maintaining homeostasis against dynamic extracellular environments [4,5]. These metabolic strategies are encoded within the genomes of individual community members, accessible through advances in sequencing technologies over the past two decades. Although studies comparing community metabolic potential among metagenomes have demonstrated changes in metabolic pathway usage based on environmental geochemistry [6,7], the focus here is on broad rather than individual metabolic pathway specific deviations in whole community taxonomy and metabolic potential across physical and geochemical gradients.

For a gene to be fixed within a subpopulation of organisms in a complex community, the cognate proteins encoded by the organisms’ genomes must function within the geochemical constrains of the environment. The narrow tolerances (e.g. temperature and pH ranges) of some proteins limit the availability of potential habitats for the whole organism, impacting gene flow and, ultimately, colonization ability of the species. For example, the habitat range of photosynthesis along a hydrothermal outflow channel, defined largely by constraints imposed by temperature [8], is a functional limitation that results in a substantial difference in community composition and function, despite negligible differences in physico-chemistry on either side of this upper temperature limit on photosynthesis. Additionally, it is becoming clear that the environmental factors that limit biological function are multidimensional. From the example above, the upper temperature limit for photosynthesis has been discovered to be both pH and sulfide dependent [9-12]. This interdependence between biology and multiple interacting geochemical parameters, as exemplified by the limited distribution of photosynthesis, leads to the hypothesis that there are many additional facets of a community’s phenotype that are being shaped by the physical and chemical characteristics of an environment. It stands to reason that many geochemical limitations on a community’s phenotype have yet to be discovered—they simply aren’t so easy to follow as, for example, the appearance of photosynthetic pigments in a community—yet they may well play central roles in defining community structure and function. The overarching goal of this work is to expand upon current methods of identifying and ultimately quantifying the ecological interactions that most significantly define the structure and function of complex ecosystems.

Here, we integrate sequence data obtained by shotgun community genome sequencing approaches (metagenomics) [13,14] with tools that enable sequence clustering based on a Markov clustering algorithm [15] with BLAST homology [15-17] to categorize metagenomic reads based on evolutionary distance [18-21]. This approach offers a distinct advantage over clustering proteins based on function (i.e. Pfam or KEGG) as the latter approach potentially filters out evolutionary distance information which often extends beyond categories based on protein function [22,23]. Therefore, Markov clustering (and homology-based clustering methods, in general) provide a more direct measure of not only functional differentiation but also overall evolutionary distance among organisms [16]. By applying Markov clustering methods to multiple metagenomic datasets sequence information can be used to determine an overall evolutionary distance between whole communities. The Markov cluster based measure of evolutionary distance can be combined with geochemical analyses allowing statistical techniques including principal components analysis (PCA) and hierarchical clustering to be brought to bear in understanding the interactions between environment and community diversity.

Whole community Markov clustering techniques were first tested using metagenomic datasets gathered along the best available physical, chemical and spatial gradients presently in public databases, and subsequently expanded to include samples gathered from a broader range of environments. This study reveals that several measures of community biodiversity have strong covariance with specific physico-chemical parameters, including temperature, pH, sodium concentration and nitrate availability. A multivariate analysis (PCA) of all geochemical parameters represents clustering by bulk geochemistry and groups metagenomic sites together based on geographic location. Differences in bulk geochemistry covary strongly with community biodiversity, indicating that the composition of the microbial community inhabiting a natural system is determined by a combination of all physical and geochemical parameters of the environment.

## Result and discussion

### Validation of markov clustering methods in metagenomic analysis

Markov clustering methods were initially focused on 22 metagenomic datasets from three studies encompassing very distinct ecosystems, chosen specifically because they extend across steep physical and geochemical gradients: a hydrothermal outflow channel [24], a hypersaline microbial mat [3] and a marine depth profile [2]. These data sets allow for Markov clustering methods to be applied to natural systems with documented community structures, allowing for validation of our methods. The caveat to integrating such a broad range of environmental studies is that, for most metagenomic samples, only a few physico-chemical parameters are available (usually temperature and pH). However, our comparisons are bolstered by an inclusion of recently published biogeochemical studies of hydrothermal ecosystems, where metagenome sequencing has been coupled to detailed physical and geochemical analyses [24,25].

### Bison Pool

**Figure 1:**
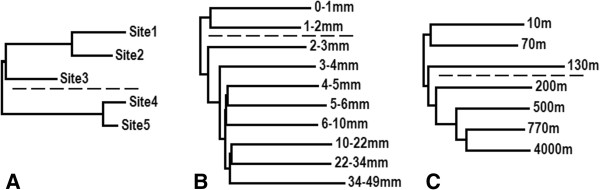
**Dendrograms based on Markov cluster dissimilarity in metagenomic datasets obtained for communities inhabiting (A) the outflow channel of ‘Bison Pool’ a vertical microbial mat profile from Guerrero Negro, Mexico (B) and an oceanic depth profile from the HOT- ALOHA (C).** Dotted lines represent major shifts in microbial community composition: **(A)** transition between chemotrophic (top) and phototrophic (bottom) metabolisms, **(B)** transition from an oxic environment near the surface (top) to a hypoxic and sulfidic environment (bottom), and **(C)** transition from photic zone (top) to aphotic zone (bottom).

The Markov clustering approach was first applied to ‘Bison Pool’, an alkaline hot spring within Yellowstone National Park, USA, where ~500 megabases of Sanger sequencing has been previously compiled from five locations along the outflow channel [24]. Sites 1, 2 and 3 were sampled from the chemotrophic portion of the outflow and sites 4 and 5 were sampled from within the photosynthetic zone. These samples span a 36°C (56.1°C to 92.1°C) temperature gradient, with concomitantly strong changes in a range of geochemical measurements such as dissolved O_2_, H_2_S, and inorganic nitrogen availability. A dendrogram (Figure 1A) based on Markov cluster analysis of these five metagenomes shows clustering of the photosynthetic sites (4 and 5) separate from the chemotrophic sites (1, 2 and 3), as would be expected based on taxonomic differences among the sites [24]. Additionally, the higher temperature chemotrophic sites cluster separately from site 3, sampled just above the highest temperature where photosynthesis occurs [11] suggesting that this “ecotone” community is transitional between high temperature chemotrophic and lower temperature photosynthetic communities.

### Guerrero Negro hypersaline microbial mats

We next applied Markov cluster to a dataset collected from Guerrero Negro, Mexico, which contains approximately 84 megabases of Sanger sequencing of community genomes sampled along millimeter depth scales through ten successive layers of a hypersaline microbial mat [3]. A cluster analysis based dendrogram representing this sample set (Figure 1B) shows clustering of the top 3 mm of the mat separate from the 4 to 50 mm samples with additional clustering of samples from similar depth ranges throughout the mat. As temperatures and pH are not reported as varying over the 49 mm depth profile we must look elsewhere for the cause of the community shifts. Commentary from this study indicates a large drop in oxygen coupled with an increase in H_2_S with depth as the driving force for microbial community changes [3]. This transition from an oxic environment to a hypoxic sulfidic environment co-occurs with a major shift in the microbial population and community metabolic strategies, captured in our cluster analysis (Figure 1B). In addition, the clustering of the top 3 mm of mat away from the bottom 46 mm likely correlates with a transition from a mixed phototrophic/chemotrophic community to one supported by chemotrophy and may be related to a shift from aerobic to anaerobic metabolism.

### HOT-ALOHA

**Figure 2:**
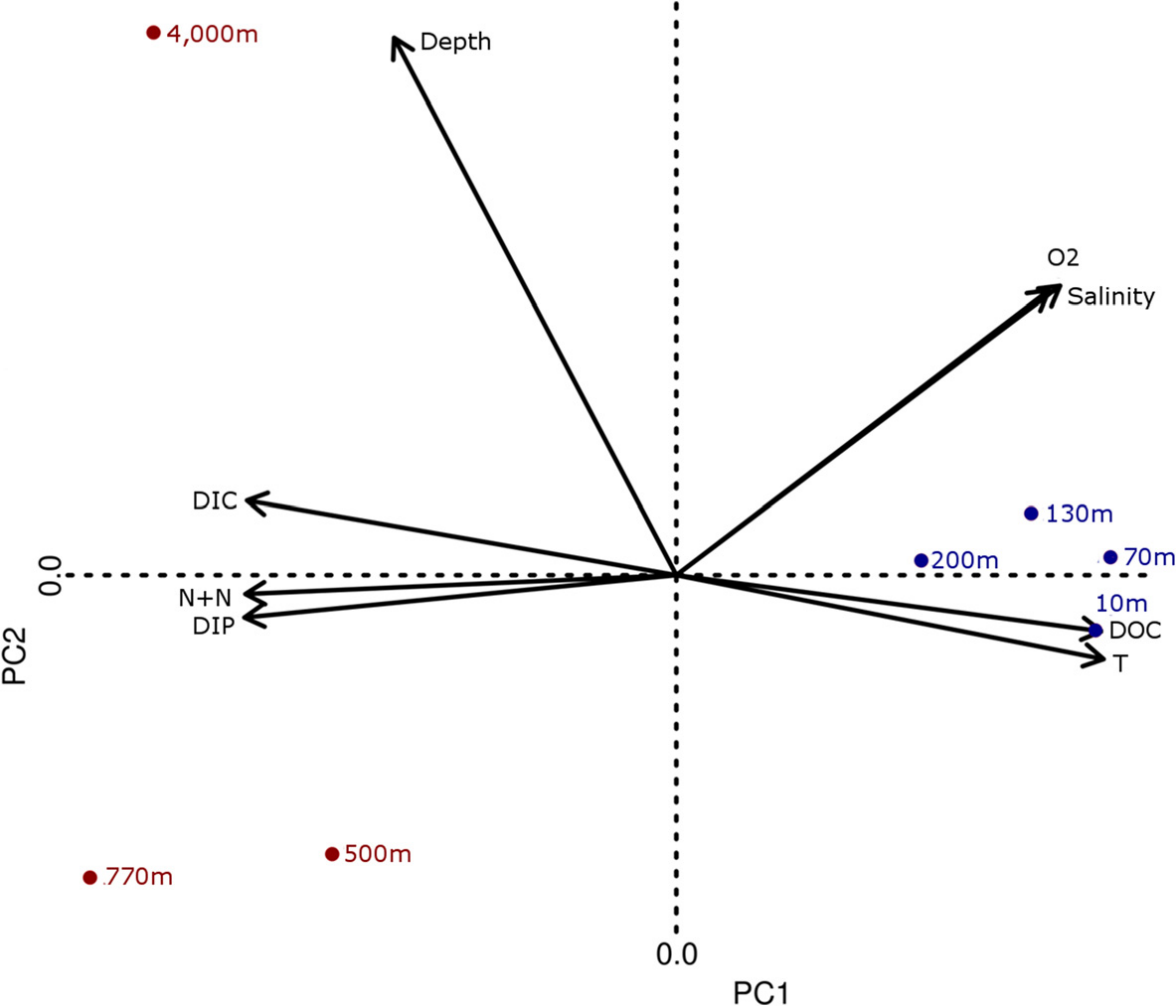
**Biplot of the two principal components (PC1 verses PC2) derived from the HOT-ALOHA geochemical data.** Sites are colored as chemotrophic (red) and phototrophic (blue).

A marine depth metagenomic profile was also included in this study as the physical and chemical characteristics of the marine water column are known to undergo changes with increasing depth [2]. Samples from the Hawaii Ocean Time-series (HOT) station ALOHA contain approximately 64 megabases of Sanger sequencing of community genomes sampled from seven depths that range from 10 to 4,000 meters [2]. A dendrogram based on Markov clustering (Figure 1C) demonstrates stratification by depth, with nearest neighbors typically coming from similar depths. Clustering occurs with the shallowest samples within the photic zone (10 m and 70 m), representing the separation of samples dominated by photosynthetic metabolisms. Principal component analysis (PCA) of reported geochemical measurements (Additional file 1: Table S1) demonstrates that the data can be reduced to two principal components (PC1 and PC2) with combined Eigenvalues explaining 96.8% of the variation (PC1 alone accounts for 85.3% of the variation). A biplot of the two principal components (PC1 verses PC2) (Figure 2) shows separation of tightly clustered photic zone depths (blue points) away from deep water depths (red points). Microbial community changes are reported along the depth gradient with surface waters including Cyanobacteria, Verrucomicrobia, Bacteroidetes and Proteobacteria while deeper waters include members of the Deferribacteres, Planctomycetes, Acidobacteria, Nitrospirae and Proteobacteria phyla (2), despite the depth vector on the biplot being orthogonal to PC1. Along PC1 temperature and dissolved organic carbon (DOC) covary and both exhibit anti-covariation with dissolved inorganic carbon (DIC), nitrite + nitrate (N + N) and dissolved organic phosphorus (DOP). On the whole, PCA demonstrates that depth, as a major component of PC2, is not a good indicator of bulk geochemistry or of community structure or function in marine samples, despite samples clearly segregating into photic and deep water clusters. PCA also suggests other unmeasured variables (most obviously, photon availability) could be driving microbial community changes and underscores an important message: missing or unmeasured physico-chemical variables directly constrain the ability to make meaningful inferences about the interaction between life and environment.

### Expanded application of markov clustering to diverse community metagenomes

#### Role of environmental variation in defining community function

**Figure 3:**
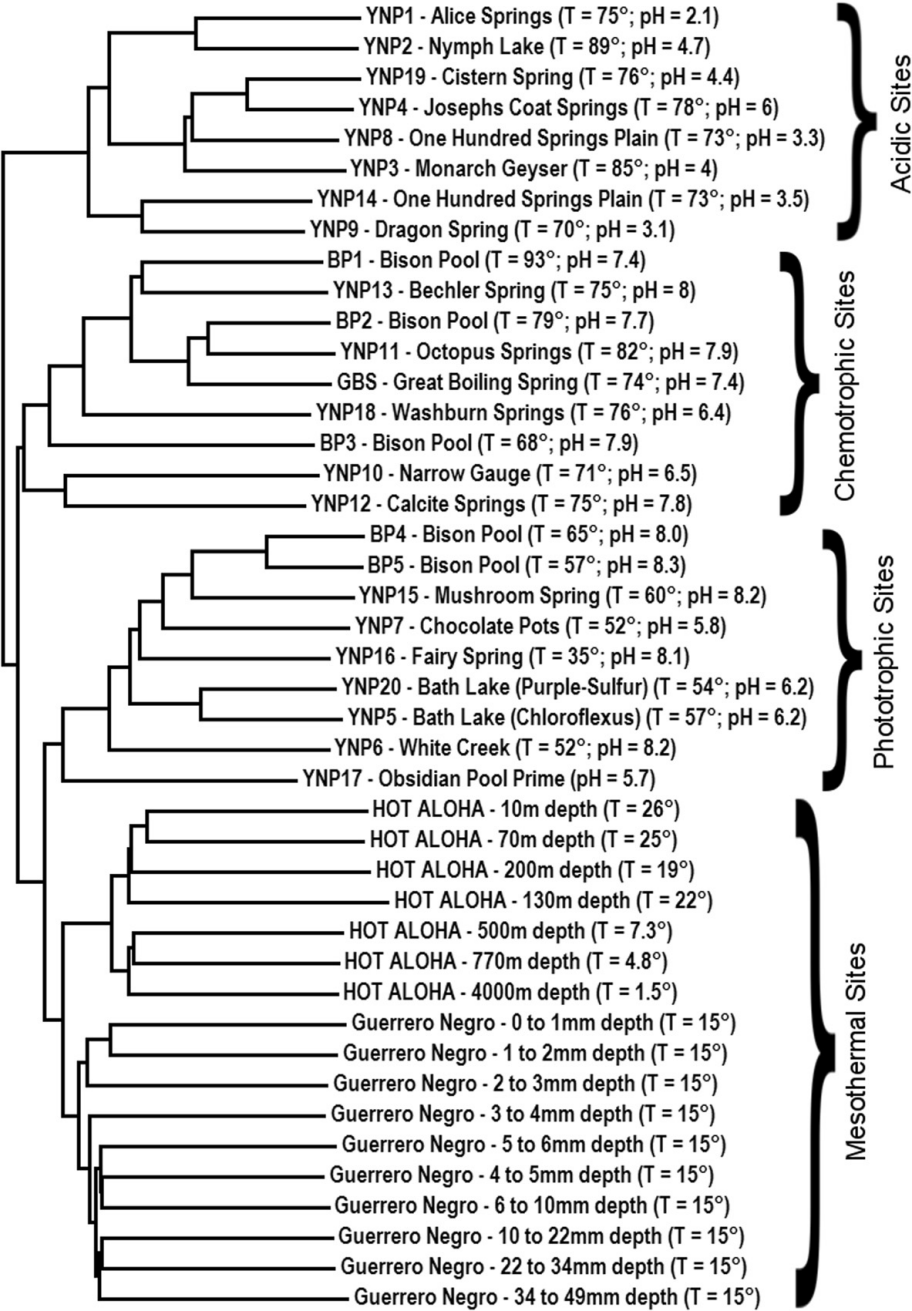
Dendrogram generated from a matrix describing the dissimilarity in Markov clusters associated with forty-three metagenomes.

The twenty-two metagenomic samples described above occur along diverse spatial and geochemical gradients and, as whole, present key opportunities to connect geochemistry to changes in biological diversity, community structure, and function. Importantly, many additional metagenomes are available that, although not purposefully sampled along continuous gradients, are useful where physico-chemical measurements were made in tandem with biological sampling. Due to the increase in available metagenomic data sets it becomes both statistically feasible and potentially very informative to correlate physical and geochemical differences to changes in the taxonomic and functional diversity of microbial communities. Correlations between geochemistry and biodiversity help identify the key geochemical parameters which shape and constrain taxonomic and functional biodiversity. Figure 3 shows a dendrogram derived from combining the Bison Pool [24], Guerrero Negro [3] and HOT-ALOHA [2] datasets with a hydrothermal sediment metagenome from Great Boiling Spring (GBS), Nevada, USA [26] and twenty additional metagenomes from Yellowstone National Park (YNP) [27-29,25]. Markov cluster analysis of this forty-three metagenome dataset shows a clear separation of hydrothermal and mesothermal sample sites, most notably the separation of the mesothermal Guerrero Negro and HOT-ALOHA sites from the hydrothermal YNP and GBS sites. Note also the temperature segregation within the hydrothermal samples: the lower temperature (phototrophic) YNP sites, including White Creek, Chocolate Pots, and Bison Pool sites 4 and 5 all cluster closest to the mesophilic sites, although the high temperature (chemotrophic) YNP and GBS sites cluster separately. A temperature dependent pattern of clustering due to functional variation between sites is intriguingly similar to the temperature dependent photosynthetic fringe mentioned previously. The successful clustering of whole microbial communities based on temperature differences provides an additional line of evidence supporting the utility of Markov clustering based approaches in comparative genomics analysis.

Additionally, a broad level of community segregation based on pH is evident across both GBS and YNP hydrothermal sites, with alkaline samples from Calcite Spring, Washburn Spring, Great Boiling Spring and Bison Pool clustering separately from acidic sites, including Alice Spring, Monarch Geyser and Cistern Spring. A pattern of clustering based on metabolic potential as a function of pH is consistent with previous studies conducted across spatial geochemical gradients in YNP which suggest that pH is the dominant factor shaping the diversification of bacteria and/or archaea at a taxonomic level [30]. The strong influence of pH on the taxonomic and functional composition of hydrothermal communities may reflect different adaptations to deal with acidity [30,31] or may reflect pH-dependent shifts in the energetics associated with inorganic redox couples thought to be fueling these communities [32].

The clustering of communities based predominantly on pH and temperature observed throughout Figure 3 is particularly notable in that it dominates clustering based on biological features, such as the taxonomic or metabolic compositions of communities [6,7]. For instance, the HOT-ALOHA, Guerrero Negro, and YNP datasets all include metagenomes dominated by cyanobacteria whose metabolism is driven by oxygenic photosynthesis, yet clustering of these photosynthetic communities by inorganic factors suggests they have evolved on trajectories optimizing their genomes for conditions specific to each of these environments.

**Figure 4:**
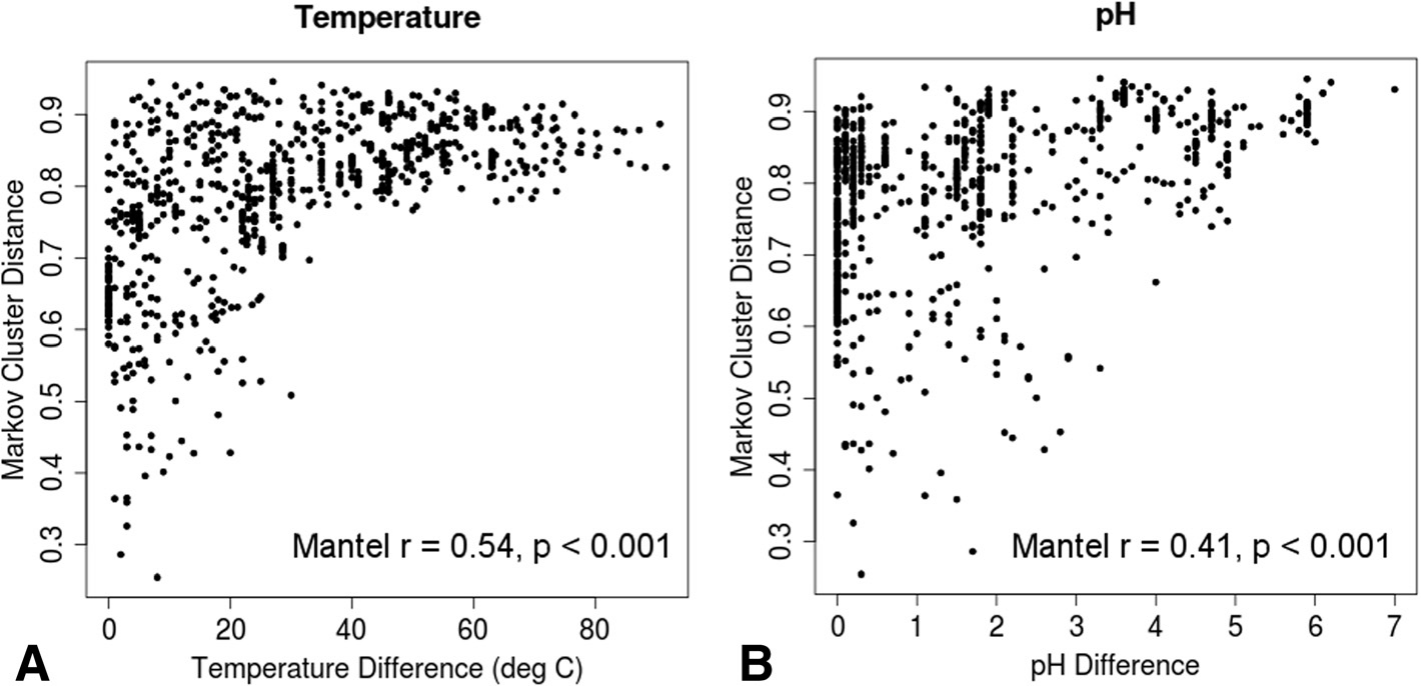
Plots of Markov cluster distance for forty-three metagenomes as a function of temperature (A) and pH (B).

Markov cluster-based evolutionary distances were plotted against temperature (Figure 4A) and pH (Figure 4B) for all pairwise comparisons among the forty-three metagenomes included in this study. Mantel tests [33] show temperature correlates with Markov distance with a Mantel r value of 0.54 (p < 0.001) and pH correlates with a Mantel r value of 0.41 (p < 0.001). Although both results show high correlations when compared to other ecological studies using Mantel r values [34-36], it is important to note that the relationships shown in both plots are clearly nonlinear. This nonlinear relationship suggests “envelopes” of allowable space demonstrating that large temperature and/or pH differences drive concomitantly large evolutionary divergences and that communities inhabiting similar temperature and pH ranges are not necessarily evolutionarily related. Certainly organisms and communities have adapted to physico-chemical extremes many times throughout the history of life, and finding unrelated communities occupying similar temperature and pH ranges supports the notion that those adaptations often occur through novel, independent, evolutionary strategies and are not simply the result of adaptation to a small set of environmental variables.

Correlations between microbial community evolutionary divergence and temperature and pH invited deeper exploration of the extensive physical and geochemical data available for some of these metagenomes, in particular a subset of twenty-two metagenomes sequenced as part of several studies of YNP hydrothermal ecosystems (Additional file 2: Table S2) [24,27-29,25]. Physical and geochemical metadata includes measurements of temperature, pH, sodium, potassium, calcium, aluminum, iron, magnesium, chloride, phosphorus, silicon, boron, arsenic, zinc, manganese, dissolved oxygen, sulfate, nitrate, sulfide, dissolved organic carbon (DOC) and dissolved inorganic carbon (DIC).

To test for correlation between evolutionary distances and geochemistry, Mantel tests [33] were performed between all geochemical parameters and Markov cluster-based evolutionary distances (Additional file 3: Figure S1 and Additional file 4: Table S3). Several parameters showed slight to moderate correlations [34-36] with evolutionary distances, including chloride (Mantel r = 0.198, p = 0.007), zinc (Mantel r = 0.199, p = 0.010), DIC (Mantel r = 0.201, p = 0.018) and silicon (Mantel r = 0.118, p = 0.045). Notably, the parameters showing strongest correlation were, once again, temperature (Mantel r = 0.376, p = 0.001) and pH (Mantel r = 0.484, p = 0.001). These results reiterate the strong influences temperature and pH have on microbial community evolutionary distance as compared to other geochemical parameters. Importantly, the lack of correlation of Markov cluster distance with some geochemical analytes does not imply lack of a relationship; because these analyses cluster entire metagenomes, the influence of physico-chemistry on individual enzymes and pathways—many of which are known to be strongly dependent on environmental conditions—is, in effect, averaged out.

A covariance matrix based on the twenty included geochemical parameters was used as the basis for a principal component analysis (PCA) of site geochemistry with the Eigenvalues for the first three principal components (PC1, PC2 and PC3) accounting for 61% of the geochemical variation among the twenty-two YNP sites. An overall geochemical distance between YNP sites was calculated by determining the Euclidean distance between YNP sites in (PC1, PC2, PC3) space. A Mantel test was then performed between the overall geochemical distance and the Markov cluster based evolutionary distance for all YNP sites finding a Mantel r value of 0.3861 (p < 0.001). Although this correlation is weaker than temperature or pH when analyzed individually, a plot of overall geochemical distance verses Markov cluster distance does not display the “envelope” seen in temperature and pH plots. Unlike the “envelope” seen with temperature and pH the overall geochemistry plot is void of points at high community evolutionary distance and low geochemical difference (upper left) indicating that substantially different microbial communities do not inhabit environments with overall similar geochemistry. PCA demonstrates that when analyzed together many site geochemical parameters act in concert to influence the microbial community populating a natural environment. Additionally, PCA hints that the strong correlation with pH might not be due to the concentration of H^+^, but to the effect pH has on the speciation of other compounds and the energetic favorability of using these compounds in microbial metabolisms [32].

#### Role of geochemical variation in defining community biodiversity

Finally, we used multivariate techniques to investigate which geochemical parameters most strongly amplify or constrain microbial community diversity. Because biodiversity can be defined quite differently depending on the context and scientific field [37,38], we chose three distinct measures of biological diversity to measure and correlate with environmental metadata. These diversity measurements include: taxonomic diversity (derived from genera counts within each metagenome), functional diversity (derived from metabolic enzyme category (EC) counts within each metagenome), and community complexity (derived from Markov cluster counts within each metagenome). These three measures of diversity were correlated with the twenty geochemical parameters described above (Table 1). Covariance (r from 0.5 to 1) is shaded black and anti-covariance (r from −1 to −0.5) is shaded grey. This analysis shows temperature anti-correlating with genera counts (r = −0.59) and EC counts (r = −0.57) while pH correlates with genera counts (r = 0.71), EC counts (r = 0.62) and Markov cluster counts (r = 0.63). The covariance matrix suggests that low temperature alkaline environments promote community biodiversity whereas high temperature acidic environments constrain biodiversity. Additionally, Markov cluster count is correlating with sodium (r = 0.50) and nitrate (r = 0.50) concentrations while genera count is anti-correlating with zinc concentration (r = −0.54); the functional significance of these relationships is not clear although the strong correlation with sodium may corroborate previous studies suggesting salinity (represented by sodium) as a predominant driver of taxonomic biodiversity [39]. Importantly, the three measurements of biodiversity correlate strongly with one another (bottom-right corner of Table 1). As genetic, functional, and taxonomic diversity are all ultimately encoded at the genetic level and subject to Darwinian evolution, this strong correlation is not surprising, but serves as reassurance that our independently derived measures of biodiversity are in-fact related.

**Table 1:** Covariance matrix for twenty geochemical variables plus three diversity metrics across twenty two metagenomic sample sites within Yellowstone National Park

	**T**	**pH**	**Na**	**K**	**Ca**	**Al**	**Fe**	**Mg**	**Cl**	**NH**^**4**^	**SO**^**4**^	**NO**^**3**^	**P**	**Si**	**B**	**As**	**Zn**	**Mn**	**S**^**2**^**-**	**O**_**2**_	**Genera**	**EC**	**Clusters**
T	1.00	−0.35	0.16	0.10	−0.26	0.17	0.02	−0.23	0.25	0.16	0.08	0.27	−0.32	0.36	0.22	0.14	0.24	0.12	−0.06	−0.41	−0.59	−0.57	−0.37
pH	−0.35	1.00	0.36	−0.10	−0.02	−0.61	−0.60	−0.05	−0.26	−0.01	−0.26	0.35	0.01	−0.21	−0.02	−0.03	−0.62	−0.33	0.05	0.48	0.71	0.62	0.63
Na	0.16	0.36	1.00	−0.06	−0.45	−0.31	−0.42	−0.49	0.65	−0.35	−0.69	0.47	−0.48	0.65	0.12	0.19	−0.05	−0.43	−0.51	0.18	0.18	0.35	0.50
K	0.10	−0.10	−0.06	1.00	0.30	−0.14	−0.12	0.30	0.28	0.06	0.16	−0.34	0.00	−0.19	0.75	0.55	0.04	−0.14	0.42	−0.26	−0.41	−0.29	−0.36
Ca	−0.26	−0.02	−0.45	0.30	1.00	−0.12	−0.10	0.99	−0.19	−0.09	0.40	−0.29	0.55	−0.64	−0.10	0.00	−0.14	−0.14	0.63	−0.25	0.07	−0.12	−0.16
Al	0.17	−0.61	−0.31	−0.14	−0.12	1.00	0.93	−0.06	−0.21	−0.09	0.26	−0.17	−0.15	0.22	−0.13	−0.16	0.38	0.19	−0.21	−0.15	−0.38	−0.27	−0.37
Fe	0.02	−0.60	−0.42	−0.12	−0.10	0.93	1.00	−0.05	−0.32	−0.08	0.25	−0.27	0.05	0.10	−0.13	−0.16	0.33	0.31	−0.20	−0.17	−0.31	−0.21	−0.33
Mg	−0.23	−0.05	−0.49	0.30	0.99	−0.06	−0.05	1.00	−0.23	−0.02	0.49	−0.27	0.52	−0.65	−0.11	−0.03	−0.13	−0.15	0.68	−0.28	0.05	−0.14	−0.18
Cl	0.25	−0.26	0.65	0.28	−0.19	−0.21	−0.32	−0.23	1.00	−0.29	−0.45	0.01	−0.40	0.65	0.25	0.30	0.40	−0.35	−0.27	−0.08	−0.33	−0.14	−0.03
NH_4_	0.16	−0.01	−0.35	0.06	−0.09	−0.09	−0.08	−0.02	−0.29	1.00	0.77	0.30	0.07	−0.19	0.30	0.20	−0.13	−0.08	0.57	−0.19	−0.01	−0.03	−0.01
SO_4_	0.08	−0.26	−0.69	0.16	0.40	0.26	0.25	0.49	−0.45	0.77	1.00	0.02	0.27	−0.44	0.09	−0.01	−0.01	−0.07	0.79	−0.36	−0.12	−0.21	−0.27
NO_3_	0.27	0.35	0.47	−0.34	−0.29	−0.17	−0.27	−0.27	0.01	0.30	0.02	1.00	−0.21	0.29	−0.10	−0.12	−0.36	−0.33	0.00	−0.01	0.36	0.44	0.50
P	−0.32	0.01	−0.48	0.00	0.55	−0.15	0.05	0.52	−0.40	0.07	0.27	−0.21	1.00	−0.60	−0.18	−0.11	−0.20	0.12	0.41	−0.24	0.23	0.06	0.04
Si	0.36	−0.21	0.65	−0.19	−0.64	0.22	0.10	−0.65	0.65	−0.19	−0.44	0.29	−0.60	1.00	0.12	0.16	0.33	−0.23	−0.59	0.15	−0.20	0.04	0.13
B	0.22	−0.02	0.12	0.75	−0.10	−0.13	−0.13	−0.11	0.25	0.30	0.09	−0.10	−0.18	0.12	1.00	0.89	−0.06	−0.05	0.15	−0.22	−0.39	−0.21	−0.21
As	0.14	−0.03	0.19	0.55	0.00	−0.16	−0.16	−0.03	0.30	0.20	−0.01	−0.12	−0.11	0.16	0.89	1.00	−0.11	−0.06	−0.02	−0.14	−0.28	−0.15	−0.07
Zn	0.24	−0.62	−0.05	0.04	−0.14	0.38	0.33	−0.13	0.40	−0.13	−0.01	−0.36	−0.20	0.33	−0.06	−0.11	1.00	0.07	−0.16	−0.20	−0.54	−0.38	−0.36
Mn	0.12	−0.33	−0.43	−0.14	−0.14	0.19	0.31	−0.15	−0.35	−0.08	−0.07	−0.33	0.12	−0.23	−0.05	−0.06	0.07	1.00	−0.23	−0.16	−0.27	−0.36	−0.34
S^2^	−0.06	0.05	−0.51	0.42	0.63	−0.21	−0.20	0.68	−0.27	0.57	0.79	0.00	0.41	−0.59	0.15	−0.02	−0.16	−0.23	1.00	−0.35	0.09	−0.04	−0.10
O_2_	−0.41	0.48	0.18	−0.26	−0.25	−0.15	−0.17	−0.28	−0.08	−0.19	−0.36	−0.01	−0.24	0.15	−0.22	−0.14	−0.20	−0.16	−0.35	1.00	0.46	0.48	0.42
Genera	−0.59	0.71	0.18	−0.41	0.07	−0.38	−0.31	0.05	−0.33	−0.01	−0.12	0.36	0.23	−0.20	−0.39	−0.28	−0.54	−0.27	0.09	0.46	1.00	0.91	0.87
EC	−0.57	0.62	0.35	−0.29	−0.12	−0.27	−0.21	−0.14	−0.14	−0.03	−0.21	0.44	0.06	0.04	−0.21	−0.15	−0.38	−0.36	−0.04	0.48	0.91	1.00	0.92
Clusters	−0.37	0.63	0.50	−0.36	−0.16	−0.37	−0.33	−0.18	−0.03	−0.01	−0.27	0.50	0.04	0.13	−0.21	−0.07	−0.36	−0.34	−0.10	0.42	0.87	0.92	1.00

Covariance between 0.5 and 1 are shaded black and anti-covariance between −1 and −0.5 are shaded grey.

**Figure 5:**
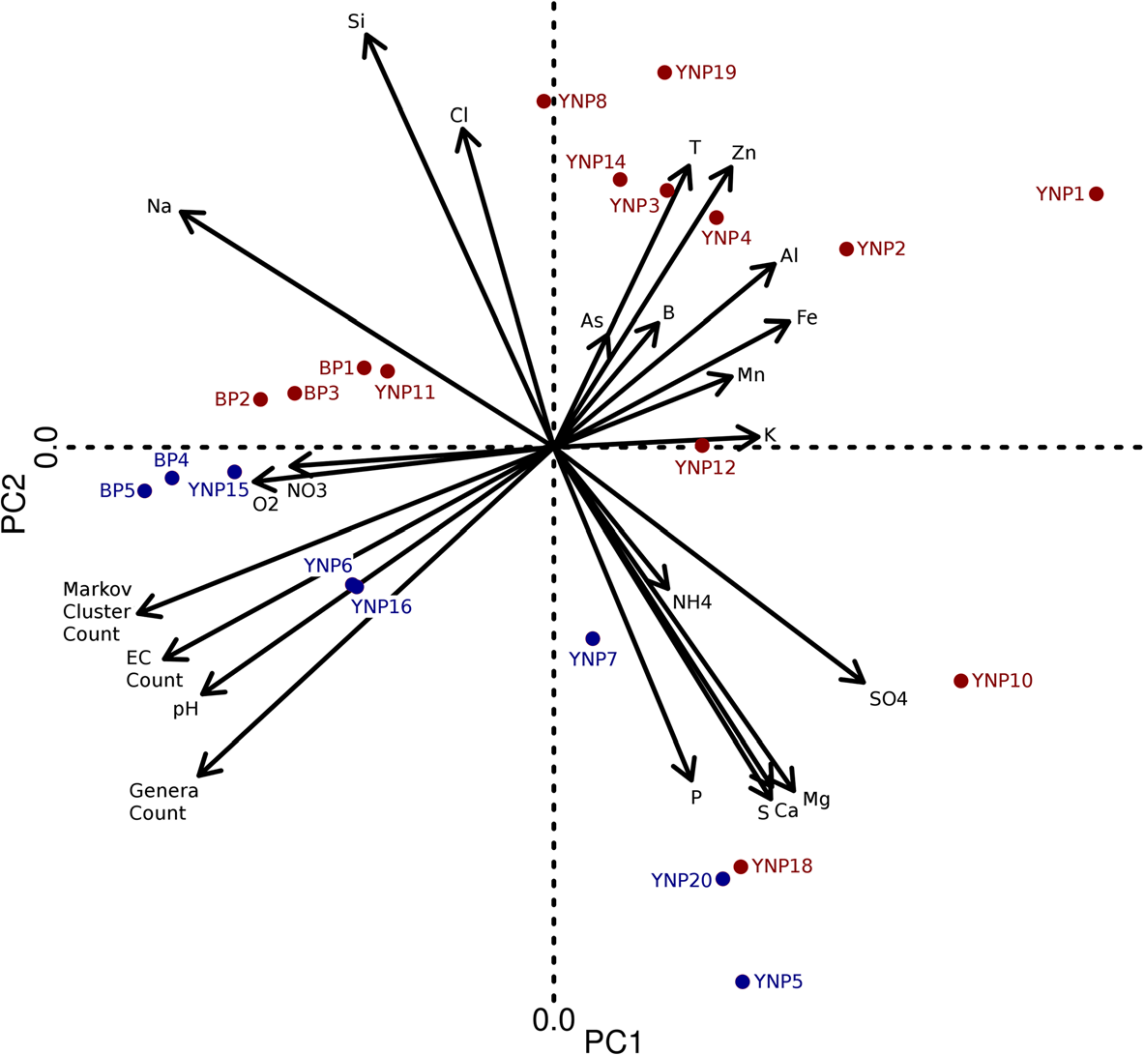
**Biplot generated from a principal component analysis (PCA) of the twenty-two YNP sites with individual sample sites and diversity metrics depicted.** Sites are colored as chemotrophic (red) and phototrophic (blue).

A biplot (Figure 5) generated from a PCA of the geochemical and diversity correlation matrix shows where the metagenomic sites lie within physico-chemical and biodiversity space. Archaeal-dominated sites (YNP 1, 2, 3, 4, 8, 14 and 19) populate the upper right quadrant of the biplot, hinting that the geochemical parameters associated with these sites exclude bacterial life that lack functional adaptations to inhabit these springs [40]. The photosynthetic mat samples collected from the Lower Geyser Basin (BP 4, 5, YNP 6, 15 and 16) show clustering, but separate from the photosynthetic mats found at Mammoth hot springs (YNP 5 and 20). Aquificales-dominated sites (YNP 10, 11, 12 and 13) do not cluster with each other, but instead cluster based on geographic proximity and geochemical similarity. For instance, YNP 11 (Octopus Spring) clusters with Bison Pool site 1; both springs are alkaline and are proximal geographically. Likewise, YNP 14 (One Hundred Springs Plain) clusters with other Norris Geyser Basin springs such as YNP 3 (Monarch Geyser). Bison Pool (BP) sites illustrate the strengths of PCA for correlating this multidimensional dataset: all five BP sites are in the same region of the biplot due to the overall similar geochemistry among sites and, further, are aligned in a linear fashion parallel to the temperature vector (due to the 32°C temperature gradient along the outflow). The placement of similar sites on the PCA biplot illustrates the predictive power of PCA as implemented here; one could reasonably predict where a new site might plot based on measurements of only a handful of well-chosen biological and physico-chemical parameters.

## Conclusions

Markov cluster based comparisons of metagenomes coupled with multivariate analyses identified many key physical and geochemical parameters which are responsible for shaping microbial community composition, function, and complexity. Most metagenomic datasets include very limited (or no) environmental metadata; here we focused on a subset of metagenomes with detailed measurements of pH and temperature, and a subset of these (from hydrothermal systems) with 18 additional geochemical measures that could be compared. Our analysis supports the strong role that pH and temperature play in influencing microbial community composition and function, accounting for the highest average Mantel correlations (0.48 for pH and 0.38 for temperature) to evolutionary distance between metagenomes. Importantly, upon the inclusion of additional geochemical parameters it was found that the availability of carbon compounds as well as micronutrients such as iron and zinc all correlate (or anticorrelate) with diversity measures. Multivariate analyses suggest that these biology-environment interactions are multidimensional: techniques integrating many physical and chemical measurements performed as well as or better than nearly all of the individual parameters at predicting differences in biodiversity. This demonstrates that the parameters typically measured as part of metagenome studies (temperature, pH, depth) can be substantially improved upon in attempts to explain or predict biological variability as a function of environmental dynamics.

Finally, this analysis lays the groundwork for predicting community metabolism and various metrics of diversity based on site geochemistry. For example, PCA analysis of YNP community metagenomes and bulk geochemistry can predict biological properties of an unknown site based on geochemistry, and vice versa. Future metagenomic studies can continue to improve the resolving power of these predictions simply by including a small number of relatively straightforward measurements of physical and geochemical conditions along with biological sampling. This study represents an important advance toward predictive understanding of biology-environment interactions, and a compelling justification for coordinating environmental/geochemical measurements in –omics-enabled studies of natural environments.

## Methods

All metagenomic datasets were downloaded as inferred amino acid sequences from the Joint Genome Institute Integrated Microbial Genomes with Microbiome Samples (JGI IMG/M) [26] web server. All metagenomic datasets were combined into a single FASTA file and compared using a complete all-verses-all NCBI BLAST [41]. BLAST results were then parsed to hits with e-values better than 10^−40^. Parsed BLAST results were fed into the mcl [16] Markov clustering algorithm using an inflation value of 1.2. The mcl algorithm generates a network where nodes represent individual genes or proteins and the edges between them are weighted based on some measure of homology (here, BLAST e value, though in principle any homology score can be used). The heuristic then performs Markov walks across this network—quasi-random walks between nodes whose probability of traversal depends upon the strength of the edge connecting them (dependent on the homology score). Network edges are strengthened or weakened based on the number of traversals during each iteration, with the inflation parameter influencing how rapidly edges are strengthened and whether or not an edge is ‘severed’. This procedure of random walks followed by edge strengthening and/or culling is iterated until convergence, typically when no edges are strengthened or lost from the network. At convergence, nodes which remain connected are output as Markov clusters. BLAST e-value cut-off and MCL inflation values were chosen such as to maximize the inclusion of homologous proteins into resultant Markov clusters [16,24]. Perl scripts were written to determine the Jaccard (binary) dissimilarity between metagenomes by summing the total clusters shared by a pair of metagenomes and dividing by the total number of Markov clusters in each metagenome pair, resulting in a dissimilarity value of 0 (all Markov clusters occur in both metagenomes) and a dissimilarity value of 1 (no Markov clusters occur in both metagenomes). Perl scripts were then used to convert Jaccard dissimilarities among all metagenomes into distance matrices. Distance matrices were converted into dendrograms using the NEIGHBOR program within the PHYLIP software package [42]. Markov cluster distances were calculated as the total branch length distance between dendrogram terminal nodes (leaves) using the TreeIO module within the BioPerl [43] software package.

Perl scripts were used to generate dissimilarity matrices using all geochemical gradients (differences) between sampled locations. Additional, Perl scripts were used to generate dissimilarity matrices from the calculated Markov cluster distances among metagenomes. Mantel tests were performed in R [44] using the Vegan package [45] function “mantel”. Mantel tests were completed with the Pearson method using 1,000 permutations. Mantel test results were plotted as geochemical difference verses Markov cluster distance for all metagenome pairs with separate plots for each geochemical parameters.

Correlation matrices and PCA analyses were completed using the base package R (version 2.11.1) [44] with the raw geochemical measurements as input. Correlation matrices were calculated using the “cor” function in R using the Pearson correlation method. PCA was completed using the Vegan package [45] functions “rda“ with scaling enabled. PCA results were graphed using the “biplot” function with scaling of species and sites.

All metagenome sequences were compared to the NCBI non-redundant (nr) database and the KEGG [46] database using NCBI BLAST [41]. EC count was determined by tallying all unique EC numbers with a minimum of two hits from the BLAST versus the KEGG database. Genus counts were completed by tallying unique genus level hits from the best BLAST hit to the nr database, if one existed with a e-value better than 10^−40^. Tally was parsed to include only genera within 80th percentile of total hits, allowing genera with very low counts to be excluded from the analysis. Markov cluster counts were a tally of the number of Markov clusters within a metagenome.

Perl scripts developed for use in this study are freely available from the study’s coauthors.

### Availability of supporting data

The data sets supporting the results of this article are available in the US Department of Energy Joint Genome Institute Genome Web Portal repository, 2009439003, 2009439000, 2010170001, 2010170002, 2010170003, 2014031002, 2015219001, 2014031003, 2013843003, 2013954000, 2013515000, 2014031006, 2013515001, 2014031004, 2015391001, 2014031007, 2014031005, 2013515002, 2013954001, 2015219002, 2016842003, 2016842005, 2016842004, 2015219000, 2016842008, 2004247000, 2004247001, 2004247002, 2004247003, 2004247004, 2004247005, 2004247006, 2004247007, 2004247008, 2004247009, 2014613002, 2014613003, 2014642001, 2014642004, 2014642002, 2014642000, 2014642003, 2053563014.

## Competing interests

The authors declare that they have no competing interests.

## Authors’ contributions

Conceived and designed the experiments: EBA ESB JR. Performed the computations: EBA. Analyzed the data: EBA ESB JR. Wrote the paper: EBA ESB JR. All authors read and approved the final manuscript.

## Supplementary Material

Additional file 1: Table S1 Geochemical metadata reported with the HOT-ALOHA metagenomic datasets [2]. Click here for file

Additional file 2: Table S2 Geochemical metadata reported with the Bison Pool [24] and Yellowstone National Park metagenomic datasets [25]. Click here for file

Additional file 3: Figure S1 Plots of temperature, pH, Al, As, Ca, Cl, dissolved organic carbon, dissolved inorganic carbon, Mg, ammonium, nitrate, dissolved oxygen, K, Si, Na, Fe, sulfate, sulfide, B, P, Zn and Mn verses Markov cluster distance for twenty two metagenomes. Click here for file

Additional file 4: Table S3 Mantel test p-values and significance results from comparisons between Markov cluster distance and geochemical differences for YNP metagenomes. Click here for file
