# Supplementary figures and images for: Merging metagenomics and geochemistry reveals environmental controls on biological diversity and evolution
Source: BMC Ecol. 2014 May 28;14:16. doi: 10.1186/1472-6785-14-16 (PMC4047435; doi:10.1186/1472-6785-14-16)

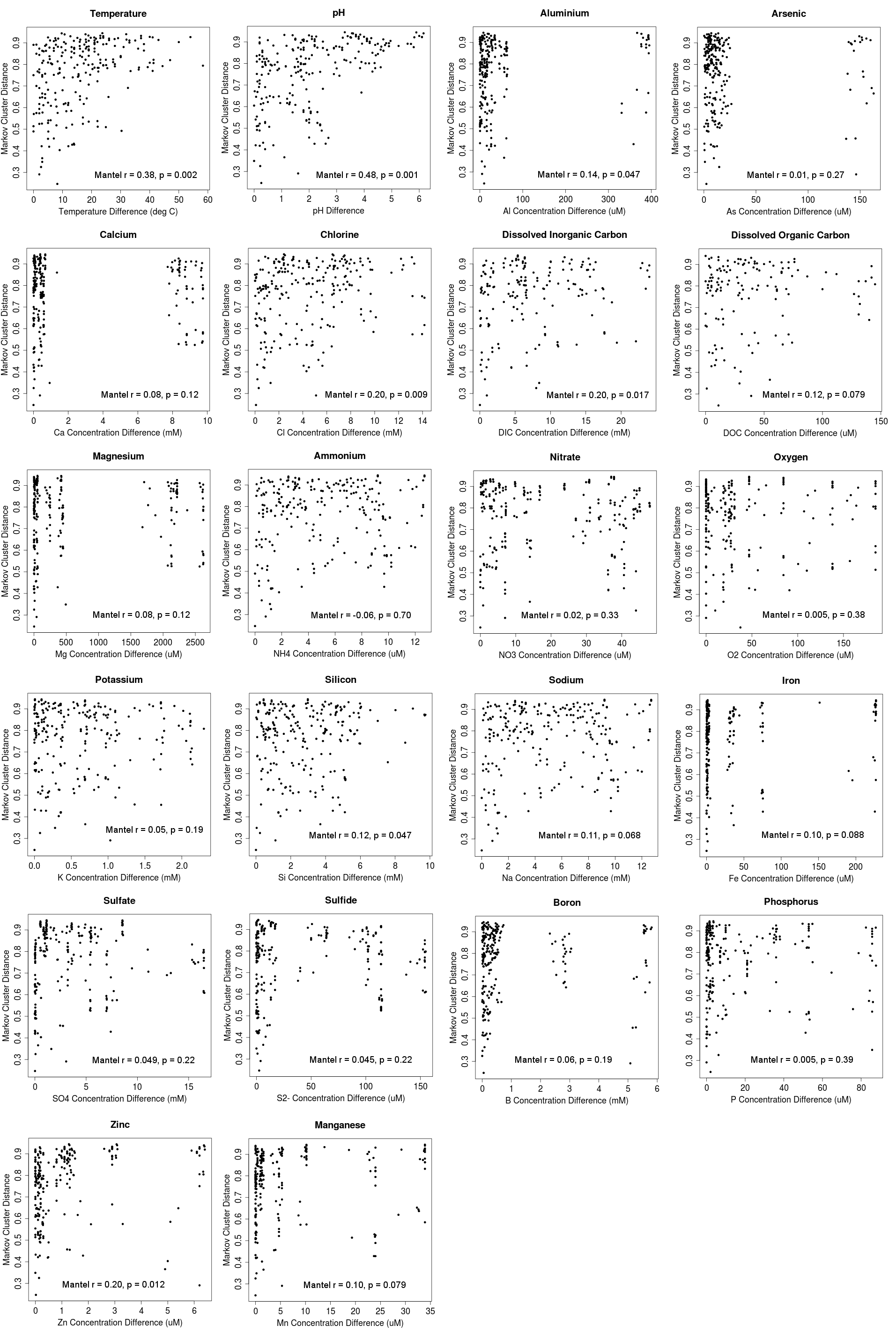

Supplement: Additional file 3: Figure S1 — Plots of temperature, pH, Al, As, Ca, Cl, dissolved organic carbon, dissolved inorganic carbon, Mg, ammonium, nitrate, dissolved oxygen, K, Si, Na, Fe, sulfate, sulfide, B, P, Zn and Mn verses Markov cluster distance for twenty two metagenomes. [file 1472-6785-14-16-S3.tiff]
